# To the Illinois Board of Health

**Published:** 1881-04

**Authors:** 


### ARTICLE X.

TO THE ILLINOIS STATE BOARD OF HEALTH:

*Gentlemen*—Your undersigned committee, appointed to report requirements of good standing for medical colleges, and conditions of complaint against medical colleges, respectfully report that the committee addressed to many leading members of the medical profession, and to the medical colleges of the Union, a circular, making the following inquiries:

1. In the present state of medical science and education in this country, what preparation is, and ought to be, required for admission to the lectures of a medical college to entitle it to good standing?

2. On what branches of medical and cognate science ought courses of lectures to be provided, and what length of course on each, to entitle the college to "good standing?"

3. What requirements as to full attendance, readings and quizzes, or other examinations, occasional or final, ought to be maintained in such colleges?

4. What attendance on lectures, as to time, number of terms or courses, and intervals between courses, are, or ought to be, required by such colleges as conditions of graduation?

5. What other conditions of graduation—such as hospital practice, or practice under preceptors, attendance on clinics and dissections—are, and ought to be, required for graduation?

To this circular eighty-seven replies were received from officers of medical colleges and medical societies and from eminent physicians. The medical colleges also promptly sent their annual announcements or circulars, and many other documents were furnished the committee touching the matters involved in their questions.

The letters, which are herewith presented to the Board, express much interest in the subject under discussion, and evince a deep and general desire for improvement in medical education in this country. It is evident that the members of the medical profession are ready to welcome and sustain all wise efforts to advance the standard and increase the thoroughness of medical instruction. The committee regret that the proper limits of their report will not permit them to quote at length from the letters received.

The annexed tabular statement, drawn from the annual announcements, will show the present usages of the medical colleges in the several respects involved in our inquiries. The letters accompanying the announcements often express a desire and purpose to introduce reforms as soon as the concurrence of other colleges will permit. This is especially true in regard to the preparatory studies to be required of candidates for admission to the lecture courses. The failure to require adequate preparation before entering upon professional studies is doubtless one of the most serious and even fatal defects in American medical education. As students usually begin their studies with private preceptors, the remedy lies first with those who consent to act as such preceptors. Let them strenuously advise all candidates to make a liberal and thorough scholastic preparation, and refuse to receive any who are destitute of such preparation, and the reform would be well begun. But the failure of private preceptors to perform this obvious duty cannot exonerate medical colleges from the responsibilities which, by implication, they voluntarily assume. The committee are glad to report that so many of the leading medical colleges have now announced preliminary examinations as conditions of admission, in accordance with the vote of the American Medical College Association, that we are justified in including such examinations in our requirements of marks of "good standing."

After a careful comparison of the usages of the colleges, and of the opinions of the profession, the committee offers the following scheme of requirements, and recommends its formal adoption by the Board as its definition of the colleges whose diplomas shall be hereafter accepted by it:

### MINIMUM REQUIREMENTS FOR A MEDICAL COLLEGE TO BE HELD IN "GOOD STANDING."

I. *Conditions of Admission* to lecture courses:
   1. Credible certificates of good moral character.
   2. Diploma of graduation from a good literary and scientific college, or high school; or, lacking this,

3. A thorough examination in the branches of a good English education, including Mathematics, English Composition and Elementary Physics or Natural Philosophy. This provision will not be required before the close of the lecture sessions of 1882–83.

II. *Branches of Medical Science* to be included in the course of instruction:

1. Anatomy.
2. Physiology.
3. Chemistry.
4. Materia Medica and Therapeutics.
5. Theory and Practice of Medicine.
6. Surgery.
7. Obstetrics and Gynæcology.
8. Hygiene and Sanitation.
9. Medical Jurisprudence.

III. *Length of Regular or Graduating Courses:*

1. The time occupied in the regular courses or sessions from which students are graduated shall not be less than five months, or twenty weeks, each.
2. Two full courses of lectures, not within one and the same year of time, shall be required for graduation with the degree of Doctor of Medicine.

IV. *Attendance and Examination*, or Quizzes:

1. Regular attendance during the entire lecture courses shall be required, allowance being made only for absences occasioned by the student's sickness, such absences not to exceed 20 per centum of the course.
2. Regular examinations, or quizzes, to be made by each lecturer or professor daily, or at least twice each week.
3. Final examinations on all branches to be conducted, when practicable, by other competent examiners than the professors in each branch.

V. *Dissections, Clinics and Hospital:*

1. Each student shall have dissected during two courses.
2. Attendance during at least two terms on clinical and hospital instruction shall be required.

VI. *Time of Professional Studies* before graduation shall not be less than three full years, including the time spent with a preceptor, attendance upon lectures or at clinics and in hospital.

VII. The college must show that it has a sufficient and competent corps of instructors, and the necessary facilities for teaching, dissections, clinics, etc.

### REMARKS ON REQUIREMENTS.

It is a matter of regret that the Board is not at liberty to adopt the practice of the best and most advanced institutions as its standard. It can only express its desire to see improvements made as rapidly as the conditions of the colleges and the public sentiment will permit.

The addition of Latin, Botany and some other branches to the preliminary examinations ought to be made as early as is practicable.

The committee also would be glad to see other important specialties of medical science and art added to the courses of required education.

### CLOSING REMARKS.

While submitting the above as the committee's conclusion upon the matter referred to them, we cannot refuse to express our concurrence in the general desire h ld by so many members of the medical profession for an early and large advance in the standards and courses of medical education in this country. Among the improvements most frequently suggested, and evidently of high importance, the committee note the following:

1. The requirement of adequate preparatory studies for admission to the lecture courses of the medical colleges. It is too shamefully true that at present many students are admitted to these lecture courses whose illiteracy prohibits their profiting by the instruction given, excepting in the narrowest limits, and precludes the possibility of their attaining such knowledge as the duties of their profession positively demand.

It would be well for the profession, and for the people, if none were accepted as students of medicine who had not graduated from a reputable college, or at least a good high school.

Natural talent and aptitude may go far toward fitting a man for any calling; but no talent can take the place of thorough education in a profession where such large fields of knowledge are to be mastered, and so many and such important judgments are to be constantly and promptly formed. Such a familiar knowledge of good English and of professional language as will enable the student to comprehend promptly and precisely the lectures to which he listens, seems too obvious a requirement to need argument, and such a knowledge cannot be gained save by years of training or extensive reading.

But it is not enough that the medical student shall merely comprehend what he hears; he must be able to weigh and judge for himself the importance of the facts, and the value of the theories which are offered.

There is a palpable absurdity in expecting to make skillful physicians of illiterate students by mere dint of reading them lectures, even when accompanied by quizzes and examinations. It is doubtful whether any examination of qualifications such as can be made at the crowded opening of a session can be relied upon to assure the requisite preparation for admission. The diploma of a high school, or, better still, of a college, would be far safer evidence of the candidate's fitness.

2. The committee heartily concur with the "American Medical College Association" in its statement that "An adequate medical education is such as gives to the student a fair practical knowledge of all the branches of medical science, and a mental discipline sufficient for the proper use of such knowledge in the practice of the medical art. A practical knowledge of these various branches necessitates demonstrative teaching and personal manipulation, which can be provided, in an adequate degree, in medical colleges only."

We would add to this obvious statement that the study of theories alone can produce nothing but theoretical knowledge. Practice only can teach practical knowledge. Abstract study may discipline the mind; but skill in any art or profession can be gained only by an actual performance of the duties of that profession. The laboratory, the dissecting-room, the clinic and the hospital practice know no substitutes. The student who does not gain skill here must win it at the bedside of his patients, and often at a fearful cost.

3. The committee also concur in the claim of the American Medical Association that "Not less than three full years should be devoted to a diligent study of medicine before graduating or commencement of practice, and that at least one-half of each of these years should be spent in a proper medical college." It should be added, that a strict and regular attendance upon all the lectures should be required, and that the time professionally spent under a preceptor should be given honestly to close and earnest study.

Whoever will carefully consider the extent of the several branches of science to be mastered by the student of medicine will easily conclude that three full years will afford but scanty time for the work to be done. Indeed, it is doubtful whether any true scholar would attempt to do his work in such time without great hesitation. How inadequately it must be done, or rather how shamefully it must be neglected, by those who reduce their attendance on lectures by fully one-half, and waste in idleness their term of private study!

4. Many of our medical colleges profess to require evidence of good moral character in their candidates. The committee are of the opinion that no requirement should be more rigidly enforced. To let loose upon the community, and into the midst of its confiding families, under the sacred name of "physician" persons of vicious habits and of immoral principles, is to send destruction where we would send healing. None but the purest and most upright can safely be trusted with the prerogative and opportunity of the family physician. The medical college offers insult and injury to mankind when it receives to its classes, or arms with its diplomas, men whose worthiness is not established by the amplest testimony.

While the Board have no authority to prescribe changes or improvements in our systems of medical education, the committee believe that we should be ready to recognize the efforts of the colleges themselves, or to advance our standard as fast or as far as the lead of these colleges will permit. No injustice is done to medical schools to require of them the highest possible standard. Their honor and their true success will alike be promoted by it. We shall only fulfill our duty as a State Board of Health by promoting to the utmost that largest and most potential force in sanitary science and in public hygiene—a well-trained and thoroughly educated medical profession.

The committee ask further time to report on the other branch of inquiry referred to them: The regulations under which complaints will be received by the Board.

(Signed) JOHN M. GREGORY,<br>W. M. CHAMBERS,<br>JOHN H. RAUCH, } *Committee.*

---

SPINAL BIFIDA TREATED WITH PLASTER OF PARIS.—Dr. Lewis A. Sayre, in a clinical lecture, says that the object of mechanical treatment is simply to protect the parts from all pressure and all possible injury until the process of ossification is completed throughout the entire length of the spinal column. This he accomplishes by first slipping over the trunk a tightly-fitting knit shirt, similar to that used in applying the plaster jacket in Pott's disease or lateral curvature. Then, having the patient held in a firm position, but without being suspended, he passes a few turns of a plaster bandage around the trunk and pelois in such a manner as to cover the spinal bifida completely. After this he cuts off a piece from both the top and bottom of the shirt, and turns the remaining portions over the part covered with plaster. He then makes a few more turns of the plaster bandage outside of all, and finally, before the plaster has had time to set, presses in the plaster with the hands on both sides of the tumor, so as to make the covering more cup-shaped, and thus protect it the more completely from all pressure. He then makes a hard, artificial roof for the spinal cord and nerves, which takes the place of the normal bony one until nature supplies the deficiency. If, on account of the child's growth, other similar plaster casings are required, they can be supplied in the same manner.—*Journal of Materia Medica.*

